# A Case of Scoring Site Shift of a Novel Scoring Balloon in a Femoropopliteal Arterial Lesion

**DOI:** 10.7759/cureus.103875

**Published:** 2026-02-18

**Authors:** Keiichiro Kishikawa, Eiji Karashima, Shioto Yasuda, Takeo Kaneko

**Affiliations:** 1 Cardiology, Shimonoseki City Hospital, Shimonoseki, JPN

**Keywords:** endovascular treatment, femoropopliteal artery, ofdi, peripheral artery disease, scoring balloon

## Abstract

Using scoring balloons is an accepted strategy for vessel preparation during endovascular treatment of femoropopliteal arterial lesions, as they create controlled longitudinal incisions that facilitate vessel expansion at lower inflation pressures and reduce the risk of uncontrolled arterial dissection. We report a case of a 74-year-old man with intermittent claudication who underwent endovascular treatment using an Aperta non-slip element (NSE) percutaneous transluminal angioplasty (PTA) balloon, a next-generation scoring balloon with four scoring wires fixed to the balloon surface to prevent slippage. The balloon was inflated three times at the same axial position without shaft rotation. Optical frequency domain imaging (OFDI) demonstrated a circumferential scoring site shift, resulting in up to eight distinct scoring incisions from a four-wire device. This phenomenon was further confirmed in an in vitro experiment. The underlying mechanism was most likely related to uneven balloon rewrapping rather than a controlled or intended effect, and its clinical benefit remains unproven.

## Introduction

According to the Japanese Circulation Society and the Japanese Society for Vascular Surgery (JCS/JSVS) 2022 guidelines, endovascular treatment (EVT) strategies for peripheral artery disease are selected based on lesion location and severity, underscoring the importance of appropriate vessel preparation [1]. Scoring balloons are an established option for vessel preparation in femoropopliteal arterial lesions during EVT [2-4]. Unlike plain balloon angioplasty, scoring balloons are designed to deliver controlled, focal mechanical stress to the vessel wall by creating longitudinal incisions. This mechanism facilitates luminal expansion at lower inflation pressures and reduces the risk of uncontrolled arterial dissection.

Previous studies have reported that performing three inflations at the same position without shaft rotation using a non-slip element (NSE) transluminal angioplasty (PTA) balloon reduces the incidence of severe dissection during femoropopliteal angioplasty [2]. Optical frequency domain imaging (OFDI), an intravascular imaging modality providing high-resolution cross-sectional visualization of the arterial wall, has been used to evaluate scoring behavior and scoring site shifts associated with repeated balloon inflations [5].

The Aperta NSE PTA balloon (Nipro Corporation, Osaka, Japan) has recently been developed as a next-generation NSE PTA balloon. Compared with the original NSE PTA balloon, the Aperta device features scoring wires attached directly to the balloon surface, a design intended to prevent wire slippage during inflation, and an increased number of scoring wires from three to four while maintaining the same wire height. Although this design is expected to improve rotational stability, the possibility of uneven balloon rewrapping after deflation has not been fully investigated. To our knowledge, this is the first report describing a scoring site shift observed after three inflations using an Aperta NSE PTA balloon in a femoropopliteal arterial lesion.

## Case presentation

A 74-year-old man with diabetes mellitus, dyslipidemia, and a history of smoking presented with intermittent claudication of the left lower extremity. The ankle-brachial index of the left limb was 0.60. Angiography revealed occlusion of the left superficial femoral artery. After crossing the lesion with a 0.014-inch guidewire, the lesion length was measured as 170 mm.

OFDI revealed a 0° calcium arc, a minimal lumen area of 1.55 mm², and a reference vessel diameter of 6.3 mm. The lesion exhibited thrombotic characteristics with minimal calcification. Thrombus aspiration was attempted, but only a small amount of thrombus was retrieved.

**Figure 1:**
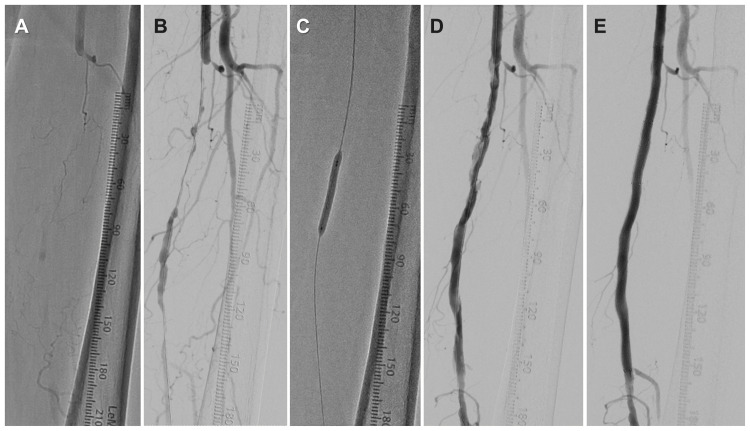
Angiography of the left superficial femoral artery lesion. (A) Pre-procedural occlusion. (B) After guidewire crossing. (C, D) After three inflations of a 5.0 × 40 mm Aperta NSE PTA balloon, showing residual stenosis and dissection. (E) After the implantation of drug-coated stents.

**Figure 2:**
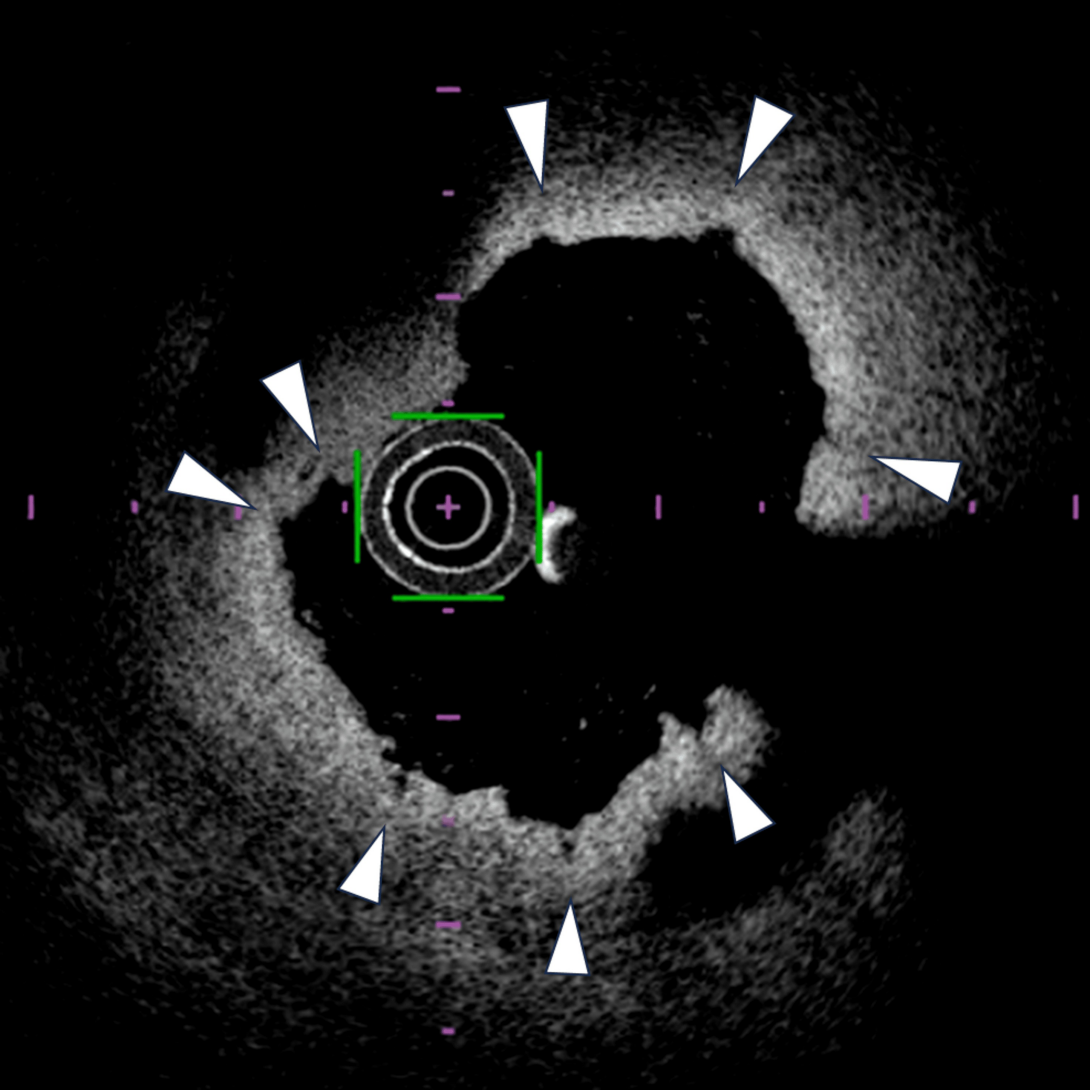
Optical frequency domain imaging after three inflations of the Aperta NSE PTA balloon. Arrows indicate individual scoring incisions. The catheter silhouette and arterial wall orientation are shown to facilitate interpretation of circumferential scoring site displacement.

A 5.0 × 40 mm Aperta NSE PTA balloon was intentionally selected for initial vessel preparation and inflated three times at the same axial position without shaft rotation. Because the balloon length was 40 mm, five sequential sessions of three inflations were performed to cover the entire lesion. Between inflations, negative pressure was manually applied using a standard deflator for approximately 5-10 seconds. Subsequent angiography demonstrated lesion dissection and residual stenosis (Figure 1). OFDI revealed up to eight distinct scoring incisions induced by the Aperta NSE PTA balloon (Figure 2).

Because significant residual stenosis, elastic recoil, and dissection persisted after additional dilation with a 6.0 mm plain balloon in the context of a thrombotic lesion, a drug-coated balloon-only strategy was considered inappropriate. Consequently, two drug-coated stents were implanted (Figure 1). After EVT, the ankle-brachial index improved to 0.94, and intermittent claudication resolved. The patient remained free from symptom recurrence for one year, and duplex ultrasound did not detect restenosis.

Experimental study

**Figure 3:**
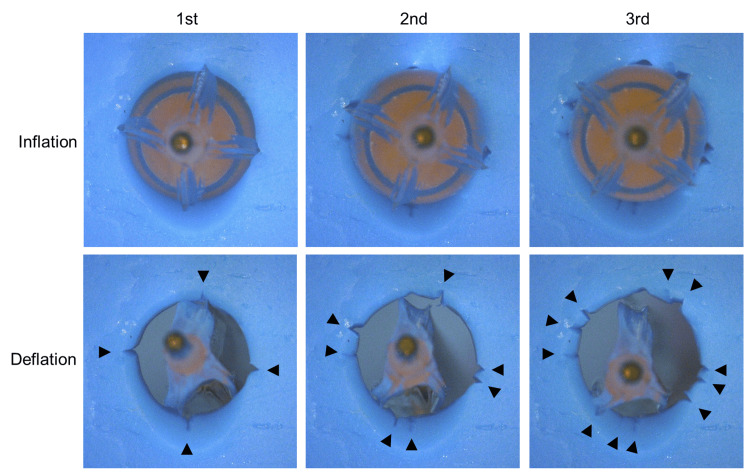
In vitro experiment of the three inflations of an Aperta NSE PTA balloon. A small hole was made in the clay, and a 6.0 × 40-mm-long Aperta NSE PTA balloon was inserted into the hole. The Aperta NSE PTA balloon was inflated to the nominal pressure and deflated three times without shaft rotation. Arrows indicate the scores induced by the Aperta NSE PTA balloon for each deflation.

To investigate the mechanism underlying the scoring site shift observed with the Aperta NSE PTA balloon, an in vitro experiment was performed (Figure 3). A soft clay model was used solely as a recording medium to visualize scoring wire contact patterns and does not replicate arterial compliance, viscoelasticity, or hemodynamic conditions. A small hole was created in the clay, and a 6.0 × 40 mm Aperta NSE PTA balloon was inserted and inflated three times at the same position without shaft rotation.

The scoring pattern was examined after each inflation. The number of scoring sites increased from four after the first inflation to seven after the second and eleven after the third inflation. Uneven balloon rewrapping was consistently observed after each deflation. This experiment was not designed to assess reproducibility or statistical variability across repeated sessions, but rather to qualitatively demonstrate the mechanical tendency toward circumferential scoring site displacement.

## Discussion

In this case, OFDI demonstrated eight distinct scoring incisions after three inflations using a four-wire Aperta NSE PTA balloon. Because the device has four scoring wires, this finding indicates a circumferential scoring site shift rather than controlled multisite scoring.

Previous studies using earlier-generation NSE PTA balloons reported that three inflations reduced severe dissection and the need for bailout stenting in femoropopliteal lesions [2]. In those devices, scoring site shifts were attributed to the absence of wire fixation [2,5]. The present case demonstrates that scoring site shift can occur even when scoring wires are fixed to the balloon surface, suggesting that uneven balloon rewrapping after deflation may play a dominant role in this phenomenon.

One of the primary objectives of scoring balloon use during EVT is the delivery of precise and predictable focal stress to facilitate vessel preparation. In thrombotic or long-segment lesions, unpredictable rotational or circumferential displacement of scoring wires may result in heterogeneous vessel injury, representing a potential loss of procedural precision rather than a benefit, and may contribute to dissection or the need for bailout stenting. In the present case, stent implantation was required because of residual stenosis, elastic recoil, and lesion characteristics, underscoring that scoring site shift did not confer an immediate clinical advantage.

This report does not provide quantitative evidence demonstrating that multisite or multidirectional scoring improves lumen gain or vessel compliance compared with stable, single-site scoring. Rather, it highlights a previously underrecognized mechanical behavior of a next-generation scoring balloon. Whether this phenomenon is beneficial, neutral, or potentially detrimental in clinical practice remains uncertain and requires further investigation.

## Conclusions

A circumferential scoring site shift was observed after three inflations of an Aperta NSE PTA balloon and was confirmed by both in vivo OFDI and an in vitro experiment. Uneven balloon rewrapping after deflation appears to be a plausible mechanism underlying this phenomenon. The clinical impact of repeated inflations with the Aperta NSE PTA balloon - whether beneficial, neutral, or potentially detrimental - remains uncertain and should be clarified in future studies.
